# Intrahepatic ectopic atypical parathyroid tumor causing primary hyperparathyroidism: a case report

**DOI:** 10.3389/fmed.2026.1780555

**Published:** 2026-03-10

**Authors:** Yuanzheng Ding, Weichao Li, Junkai Li, Zhiwei Zhang, Yuli Gao, Bo Yuan, Caixia Cheng, Jing Liu

**Affiliations:** 1 Department of Thyroid Surgery, First Hospital of Shanxi Medical University, Taiyuan, China; 2 Department of Hepatobiliary Surgery, First Hospital of Shanxi Medical University, Taiyuan, China; 3 Department of Pathology, First Hospital of Shanxi Medical University, Taiyuan, China

**Keywords:** atypical parathyroid tumor, case report, ectopic parathyroid tumor, liver, primary hyperparathyroidism

## Abstract

Primary hyperparathyroidism (PHPT) most commonly arises from cervical parathyroid pathology, with ectopic disease representing a rare entity. We report a case of PHPT diagnosed during fracture workup in a female patient who manifested persistent hypercalcemia and elevated parathyroid hormone (PTH) despite two prior cervical explorations. Cross-sectional and functional imaging, including PET/CT, localized a PTH-secreting hepatic lesion, which was confirmed by ultrasound-guided biopsy. Laparoscopic resection of the intrahepatic tumor resulted in prompt biochemical normalization. Histopathology confirmed an intrahepatic ectopic atypical parathyroid adenoma. This case underscores that refractory PHPT with persistent biochemical derangements following conventional cervical surgery should prompt consideration of rare ectopic parathyroid neoplasms with atypical histologic features.

## Introduction

Primary hyperparathyroidism (PHPT) is a common endocrine disorder characterized by parathyroid gland pathology that drives autonomous parathyroid hormone (PTH) hypersecretion, with resultant derangement of calcium-phosphate homeostasis manifesting as hypercalcemia and hypophosphatemia. Clinically, patients may present with multisystem manifestations, including renal complications (e.g., nephrolithiasis, hypercalciuria), skeletal involvement (e.g., osteoporosis, osteitis fibrosa cystica), neuropsychiatric symptoms (e.g., muscle weakness, atrophy, fatigue, depression, circadian rhythm disturbances), and cardiovascular abnormalities (e.g., valvular or myocardial calcification) (1). While most PHPT cases arise from orthotopic parathyroid lesions, this report describes the diagnostic and therapeutic challenges of a patient with PHPT secondary to an extremely rare intrahepatic ectopic atypical parathyroid tumor (2) to provide clinical management insights for similar cases.

## Case report

**Figure 1:**
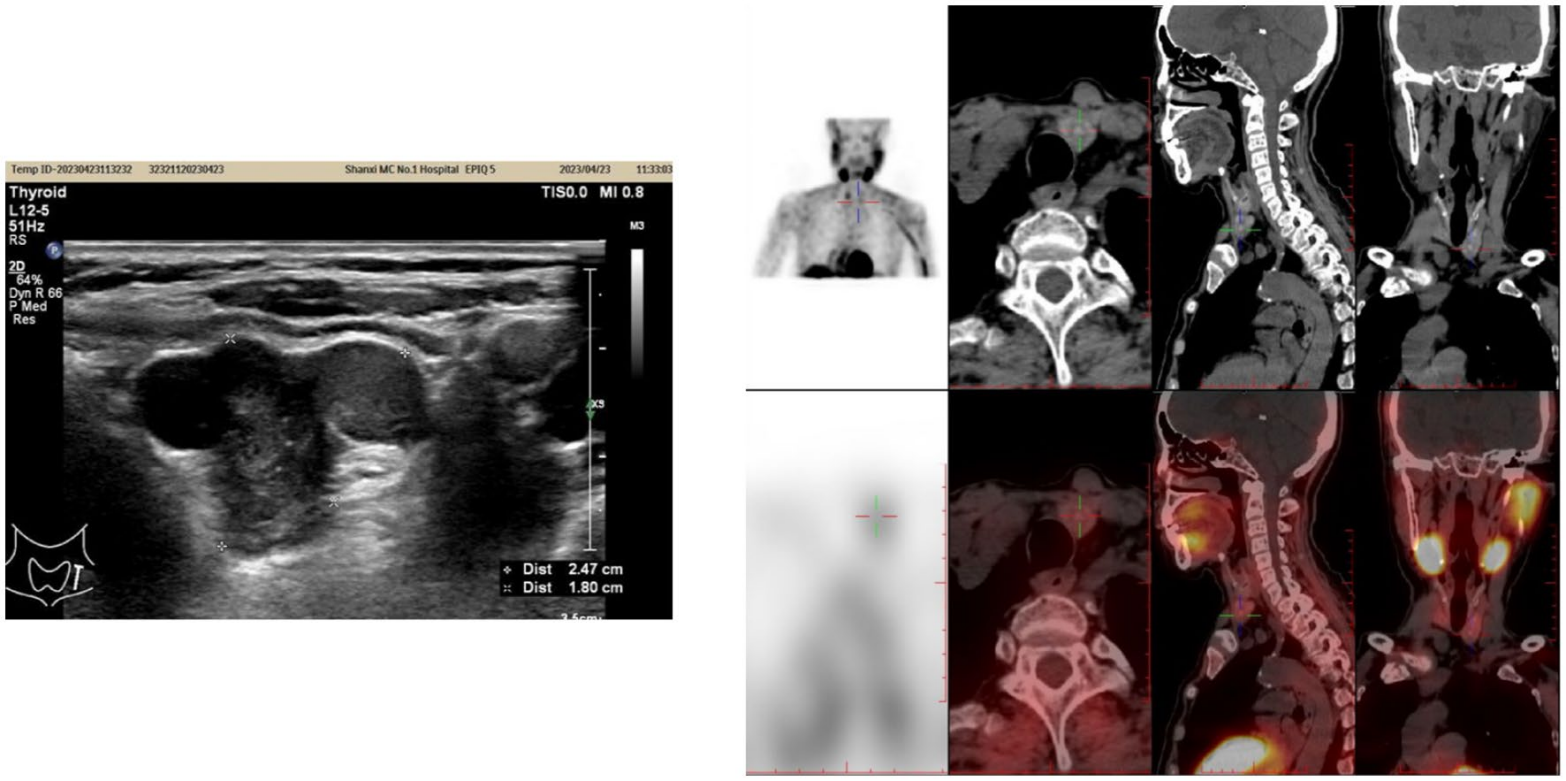
**(A)** Parathyroid color Doppler ultrasound image performed in April 2023. **(B)** ^99m^Tc-MIBI parathyroid scintigraphy image performed in June 2025.

A 78-year-old female presented with systematic fatigue and bone pain in April 2022 and was diagnosed with a parathyroid tumor accompanied by PHPT at a local hospital in December 2022 following a traumatic fracture. Surgery was recommended, but the patient declined for personal reasons. In April 2023, she was admitted to our hospital with progressive bilateral lower limb pain. Laboratory evaluation revealed markedly elevated serum PTH (1,195 pg./mL) and calcium (2.69 mmol/L). Thyroid ultrasonography (Figure 1A) showed a hypoechoic nodule, measuring approximately 2.5 × 1.8 cm, located inferior to the lower pole of the left thyroid lobe—suggestive of a parathyroid lesion or an abnormally enlarged lymph node—and a nodule in the lower pole of the right thyroid lobe (TI-RADS 4a). After precise localization by ^99m^Tc-MIBI parathyroid scintigraphy, the patient underwent resection of the right thyroid nodule and excision of the left inferior parathyroid tumor on April 23, 2023. Postoperative pathology confirmed a left inferior parathyroid adenoma and a minute focus of papillary thyroid carcinoma (<0.1 cm) in the lower pole of the right lobe. On postoperative day 1, serum PTH decreased to 61.89 pg./mL and calcium to 2.38 mmol/L. However, follow-up approximately one month later showed a rebound in serum PTH to 488.8 pg./mL, with calcium at 1.96 mmol/L. Subsequently, the patient did not adhere to regular follow-up evaluations.

**Figure 2:**
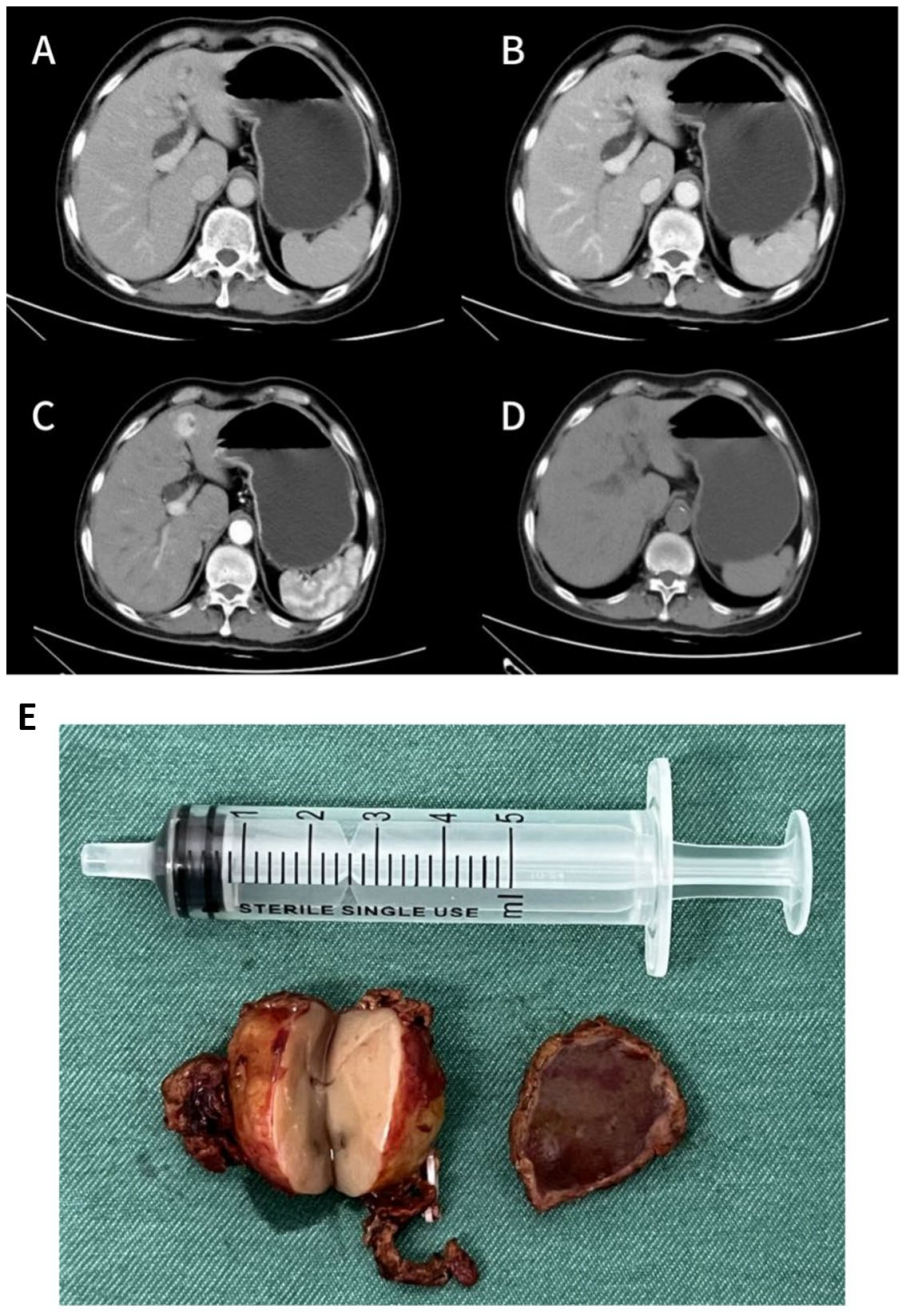
**(A–D)** Contrast-enhanced abdominal CT images performed in June 2025 (non-contrast, arterial, venous, and delayed phases). **(E)** Gross specimen of the hepatic tumor.

**Figure 3:**
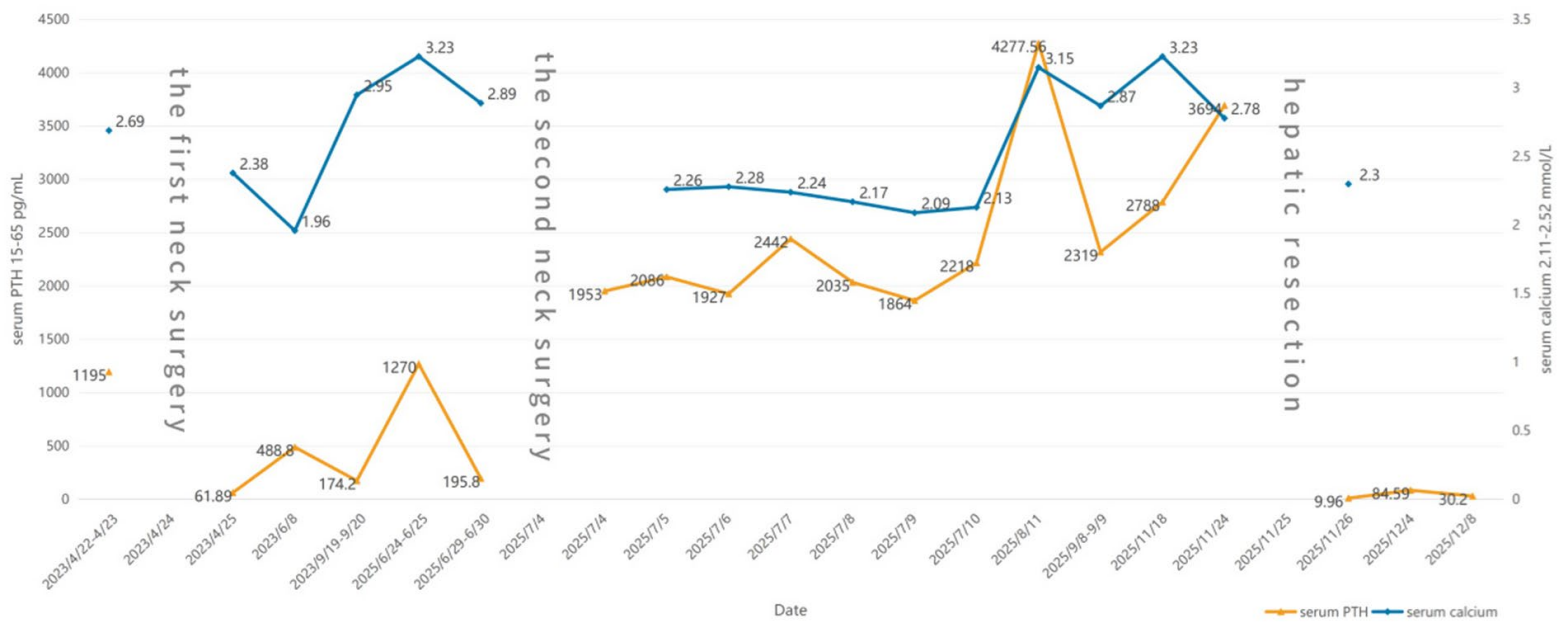
Serum PTH and calcium dynamics.

In June 2025, the patient presented with recurrent fatigue, malaise, and anorexia. Laboratory testing showed PTH of 1,270 pg./mL. SPECT/CT (Figure 1B) demonstrated a hypodense nodule (1.2 × 1.1 cm) with abnormal ^99m^Tc-MIBI uptake in the mid-lower pole of the right thyroid lobe, raising suspicion of hyperfunctioning parathyroid tissue, likely an adenoma. Additionally, contrast-enhanced CT of the neck, chest, and abdomen revealed a solid mass measuring approximately 2.7 × 1.9 cm in the left lobe of the liver (Figures 2A–D), which showed no abnormal radiotracer uptake on SPECT/CT. Given the severe metabolic consequences of uncontrolled PHPT, it was decided to prioritize surgical intervention for the parathyroid lesion. Based on previous pathology, the patient underwent right thyroid lobectomy with isthmusectomy, resection of the right inferior parathyroid tumor and right level VI lymph node dissection on July 3, 2025. Final pathology confirmed a right inferior parathyroid adenoma (PTH-positive by immunohistochemistry), and follicular nodular disease of the right thyroid lobe. Paradoxically, on the first postoperative day, serum PTH surged to 1,953 pg./mL, with calcium at 2.26. Subsequently, serum PTH remained persistently elevated at abnormal levels (Figure 3).

**Figure 4:**
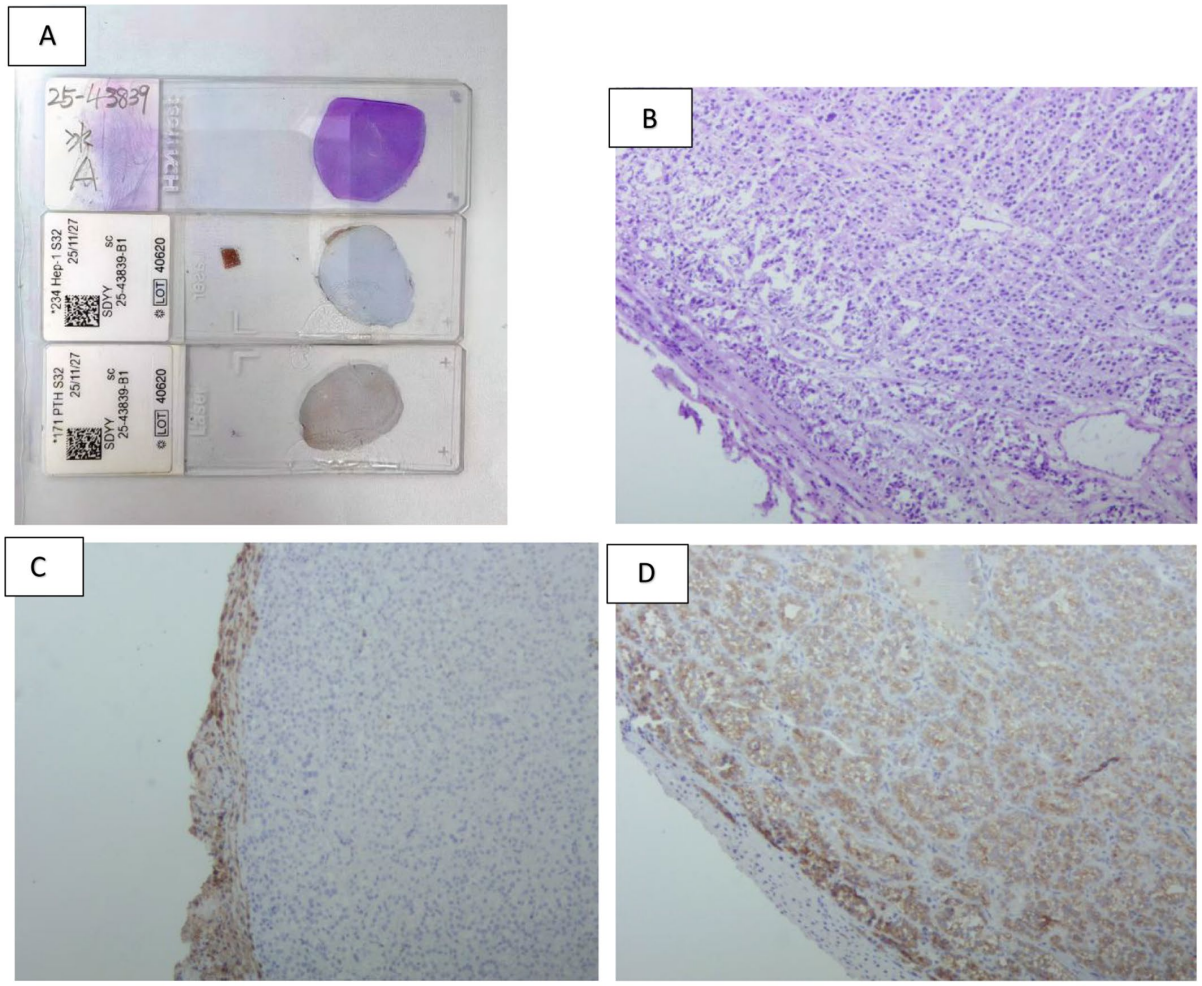
**(A)** (Top) Frozen-section HE staining of the entire tumor, showing a well-circumscribed nodular mass. (Middle) Immunohistochemical staining for HepPar-1. (Bottom) Full-section immunohistochemical staining for PTH. **(B)** HE staining at the tumor-liver interface (×100), revealing a thin fibrous capsule separating the tumor from the adjacent hepatic tissue. **(C)** HepPar-1 immunohistochemical staining at the tumor–liver interface (×100), demonstrating minimal surrounding liver parenchyma and a sharply demarcated tumor border. **(D)** PTH immunohistochemical staining at the tumor-liver interface (×100), confirming PTH expression within the tumor cells with a smooth, well-defined margin.

**Figure 5:**
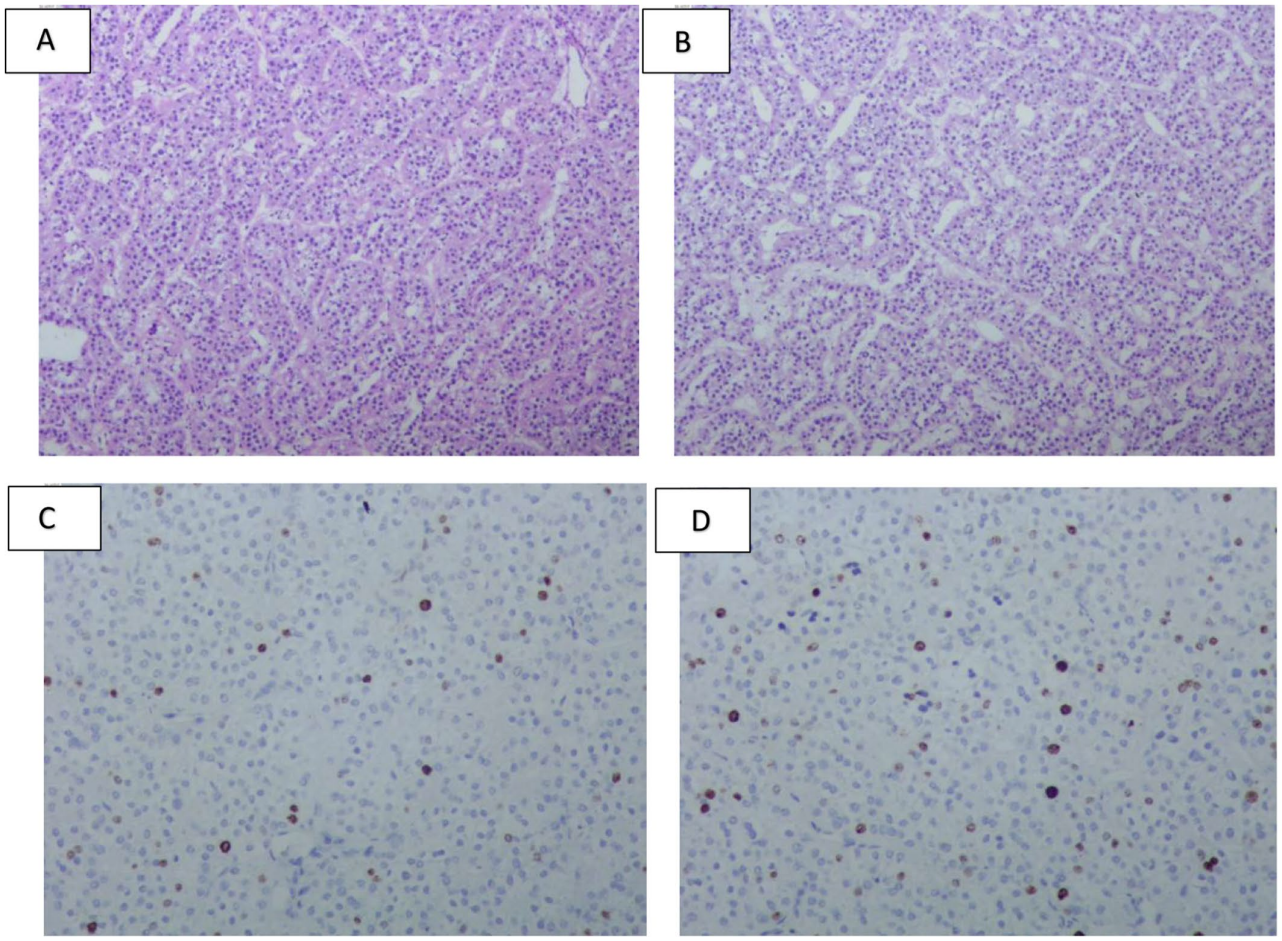
**(A,B)** HE staining (×100) illustrating an organoid architecture of the tumor cells, predominantly composed of chief cells with mild atypia, and absence of high-grade atypical foci or mitotic figures. **(C,D)** Ki-67 immunohistochemical staining, indicating a tumor cell proliferation index ranging from 2 to 5%.

Given the patient’s complex condition, an immediate multidisciplinary team (MDT) discussion was conducted, and a thorough review of preoperative examinations was performed. The CT scan revealed a mass in the left lobe of the liver as the only abnormal finding, leading to suspicion that this lesion might secrete PTH. Ultrasound-guided biopsy pathology demonstrated that the tumor cells were arranged in a nested pattern with clear cytoplasm and moderately sized nuclei. Immunohistochemical analysis showed positivity for PTH and GATA3, while markers for hepatocellular carcinoma, medullary thyroid carcinoma, renal cell carcinoma, and thyroid carcinoma were all negative, confirming the parathyroid origin of the lesion. Wild-type P53 supported its benign biological behavior, but a Ki-67 index of 5% + indicated relatively high proliferative activity. The PTH level in the biopsy washout fluid was as high as 5,000 pg./mL, further supporting the functional parathyroid origin of the hepatic lesion. Given the patient’s markedly elevated PTH levels, characterization of the hepatic lesion as benign or malignant was required to guide surgical management. Following multidisciplinary team discussion, [^18^F]AlF-NOTA-Octreotide PET/CT was performed to exclude pathologies associated with multiple endocrine neoplasia. The scan, undertaken in September 2025, demonstrated a hypodense nodule at the junction of liver segments S4 and S2 with no significant abnormal octreotide uptake, indicating a low probability of malignancy. Additional examinations ruled out other endocrine tumors or metastatic possibilities. On November 25, the patient underwent laparoscopic hepatic mass resection. Intraoperative findings (Figure 2E) showed a solid, well-demarcated, and encapsulated tumor with no local adhesion or invasion. Postoperative pathology and immunohistochemistry (Figures 4, 5) demonstrated positivity for PTH and GATA3, supporting its parathyroid origin, while classic neuroendocrine markers (Syn, CD56, INSM1, CgA) were negative. Ki-67 (2–5%) and Vimentin positivity indicated atypical features. Comprehensive analysis led to the diagnosis of an intrahepatic ectopic atypical parathyroid tumor. On the first postoperative day, serum PTH and calcium levels returned to normal (9.96 pg./mL and 2.3 mmol/L, respectively) and have remained within the normal range since (Figure 3). The patient is currently under continued follow-up.

The patient had an 11-year history of hypertension controlled with amlodipine. She has a history of multiple hospitalizations for stones and fractures, including surgery for nephrolithiasis (July 2017); common bile duct and gallbladder stone surgery (March 2018); and external fixation for a left toe fracture (2021). These urolithiasis, choledocholithiasis, and recurrent fractures are all complications associated with PHPT caused by the parathyroid tumor.

## Discussion

PHPT most commonly arises from sporadic, solitary adenomas (approximately 84% of cases), while atypical parathyroid tumors (aPT) and parathyroid carcinomas (PC) are exceedingly rare, collectively accounting for less than 2% of surgically confirmed parathyroid neoplasms (3, 4). aPT represents a borderline lesion of uncertain malignant potential, with an estimated incidence ranging from 0.5 to 4.4% (5). It shares histological features with PC—such as sheet-like or trabecular growth, cellular atypia, fibrosis, and capsular extension—yet lacks definitive criteria for malignancy, including capsular, vascular, or perineural invasion, local tissue infiltration, or distant metastasis. aPT is frequently associated with inactivating mutations in tumor suppressor genes such as CDC73 (6, 7). These tumors can cause significant biochemical disturbances, with PTH reaching up to 12 times the upper limit of normal (6).

Approximately 9–22% of PHPT cases are caused by ectopic parathyroid tumors (8), which are commonly located in areas such as the mediastinum and submandibular region. Their origin is often associated with the aberrant migration of pharyngeal pouch tissue during early embryogenesis (9). The parathyroid glands arise from the third and fourth pharyngeal pouches, while the liver develops from the hepatic diverticulum of the foregut endoderm. Residual pharyngeal pouch tissue becoming incorporated into the developing liver may lead to intrahepatic ectopic parathyroid tissue.

Surgery is an effective curative treatment for PHPT (3, 10) and precise preoperative localization of the hyperfunctioning parathyroid tumor is crucial. Currently, the combination of cervical ultrasound and ^99m^Tc-MIBI scintigraphy serves as the primary diagnostic strategy, with a combined sensitivity of 87–95% (11–14). Concordant positive findings from both exams provide a clear surgical target. However, false-negative or false-positive results may occur due to factors such as small tumor size, multifocal or ectopic lesions, expression of P-glycoprotein or multidrug resistance-associated proteins, and coexisting conditions like nodular goiter or Hashimoto’s thyroiditis (13). For suspected ectopic (especially extra-cervical) parathyroid tumors, single-photon emission computed tomography (SPECT) or hybrid SPECT/CT imaging is recommended to improve localization accuracy (15). In cases where imaging findings are equivocal, ultrasound-guided fine-needle aspiration with PTH washout measurement can serve as an adjunctive diagnostic tool (9, 16). In this case, SPECT/CT demonstrated high sensitivity for the cervical in-situ lesion. However, since the tracer ^99m^Tc-MIBI undergoes hepatobiliary clearance, the radioactive signal from the intrahepatic ectopic lesion was obscured by the high background activity of the liver parenchyma, limiting its detection. Furthermore, functional imaging modalities such as 4D-CT, [^18^F]Fluorocholine PET/CT, and [C1-^11^C]methionine PET/CT can aid in the detection of ectopic parathyroid adenomas. Specifically, [^18^F]Fluorocholine PET/CT shows high sensitivity (85–96%), and the longer half-life of [^18^F] (110 min) eliminates the need for an on-site cyclotron, making it more suitable for routine clinical imaging (16). Unfortunately, these advanced imaging techniques are not routinely employed for parathyroid tumor diagnosis at our institution.

In this case, the markedly elevated PTH level warranted differentiation from multiple endocrine neoplasia,which is a syndrome characterized by the triad of PHPT, pancreatic neuroendocrine tumors, and pituitary neuroendocrine tumors (17). To comprehensively evaluate potential neuroendocrine tumors, [^18^F]AlF-NOTA-Octreotide PET/CT was selected following MDT discussion, as this tracer demonstrates high sensitivity for detecting somatostatin receptor-positive neuroendocrine tumors (18). The imaging revealed low tracer uptake in the hepatic lesion, which aligned with the final diagnosis of a low-grade malignant potential.

This article reports a case of PHPT caused by an intrahepatic ectopic aPT. Such cases pose challenges in localization, pathological diagnosis, and surgical management due to their rare anatomical location and uncertain biological behavior. By systematically reviewing the diagnosis and treatment process of this case, we aim to deepen the understanding of this disease and to explore the associated diagnostic and therapeutic challenges and strategies. Based on the experience from this case, for patients whose biochemistry does not resolve after resection of an in-situ parathyroid adenoma, the possibility of ectopic or multifocal lesions should be considered. Establishing a MDT is crucial for comprehensive evaluation and decision-making (19). Postoperatively, serum PTH and calcium levels should be dynamically monitored to assess efficacy and detect recurrence. Given the potential recurrence risk of aPT, extended follow-up is recommended to monitor long-term patient outcomes.

## Patient perspective

With the patient’s informed consent, we have incorporated a summary of their experience. The patient described significant physical and psychological distress throughout the four-year diagnostic process. Despite previous operations, the persistence of symptoms resulted in considerable frustration and ongoing uncertainty. The conclusive diagnosis of an intrahepatic ectopic adenoma finally provided a clear explanation. By sharing this case, the patient hopes to advance medical understanding and support earlier recognition and management of similar rare conditions.

## Data Availability

The original contributions presented in the study are included in the article/supplementary material, further inquiries can be directed to the corresponding authors.
